# Do alcohol control policies work? An umbrella review and quality assessment of systematic reviews of alcohol control interventions (2006 – 2017)

**DOI:** 10.1371/journal.pone.0214865

**Published:** 2019-04-10

**Authors:** Nandi Siegfried, Charles Parry

**Affiliations:** Alcohol, Tobacco and Other Drug Research Unit, South African Medical Research Council, Tygerberg, South Africa; University of Arkansas for Medical Sciences, UNITED STATES

## Abstract

**Background:**

The 2010 World Health Organization Global Strategy to Reduce the Harmful Use of Alcohol recommends countries adopt evidence-based interventions.

**Aim:**

To update, summarize, and appraise the methodological rigour of systematic reviews of selected alcohol control interventions in the Strategy.

**Methods:**

We searched for systematic reviews across PUBMED, EMBase and The Cochrane Library in 2016 and updated in 2017 with no language limits. Two investigators independently in duplicate conducted screening, eligibility, data extraction, and quality assessment using the ROBIS tool. We categorised interventions according to the WHO recommendations, and rated reviews as at high, low or unclear risk of bias. We applied a hierarchical approach to summarising review results. Where overlap existed we report results of high quality reviews and if none existed, by most recent date of publication. We integrated the ROBIS rating with the results to produce a benefit indication.

**Results:**

We identified 42 systematic reviews from 5,282 records. Almost all eligible reviews were published in English, one in German and one in Portuguese. Most reviews identified only observational studies (74%; 31/42) with no studies from low or lower-middle income (LMIC) countries. Ten reviews were rated as low risk of bias. Methodological deficiencies included publication and language limits, no duplicate assessment, no assessment of study quality, and no integration of quality into result interpretation. We evaluated the following control measures as possibly beneficial: 1) community mobilization; 2) multi-component interventions in the drinking environment; 3) restricting alcohol advertising; 4) restricting on- and off-premise outlet density; 5) police patrols and ignition locks to reduce drink driving; and 6) increased price and taxation including minimum unit pricing.

**Conclusions:**

Robust and well-reported research synthesis is deficient in the alcohol control field despite the availability of clear methodological guidance. The lack of primary and synthesis research arising from LMIC should be prioritised globally.

## Background

Implementation of alcohol control policies of proven effectiveness will potentially lead to reduced harms from alcohol use at both individual and population levels. Such policies should ideally be based on comprehensive, high-quality and up-to-date evidence from systematic reviews of relevant interventions in which the quality of evidence is clearly graded. A systematic review identifies, appraises and synthesizes all the empirical evidence that meets pre-specified eligibility criteria to answer a given research question and provides guidance on where further evidence is required.

The use of systematic reviews forms the foundation of knowledge translation and is integral to guidelines development. Following publication of a 2007 Lancet article highly critical of the World Health Organization’s (WHO) reliance on experts instead of evidence when making global recommendations [1], WHO endorsed the use of the Grading of Evidence, Assessment, Development and Evaluation (GRADE) framework for normative guidelines development [2]. GRADE provides a method to rate the overall certainty of evidence arising from a systematic review as *high*, *moderate*, *low* or *very low*, dependent on the risk of bias, precision, consistency, and directness of the results of the included primary studies [3]. Integration of the level of certainty of evidence into the interpretation of results arising from a systematic review is a mandatory component of the GRADE approach. Inclusion of GRADE (or a similar approach) is an indicator of review quality and as such, is a key domain in the new Risk of Bias in Systematic Reviews (ROBIS) evaluation tool which allows for assessment of the quality of the methods employed in a systematic review [4].

In 2010, the WHO produced *The Global Strategy to Reduce the Harmful Use of Alcohol* listing ten interventions and policy options that countries should consider implementing based on several initiatives to collate and rate the effectiveness of each option, including whether or not a systematic review or meta-analysis had been undertaken [5]. Given the time elapsed since the publication of the *Strategy*, and the ongoing need to identify research gaps, we sought to conduct an overview of systematic reviews of alcohol control policy interventions published between 2007 and 2017. We aimed to: 1) appraise the methodological rigour of each review using the ROBIS tool [4]; 2) evaluate the utility of the ROBIS tool applied to reviews of public health interventions, specifically alcohol control; and 3) to summarise and synthesize the current evidence for each intervention type.

## Methods

We used methods for umbrella reviews recommended by the Cochrane Collaboration [6] after developing a protocol (available from authors).

### Search strategy

We developed a broad search strategy iteratively with the assistance of an experienced information specialist. The search comprised database-specific syntax and free-text terms for ‘alcohol’ and ‘alcohol consumption’ combined with terms for ‘systematic review’ and ‘meta-analysis’. We did not include terms for alcohol-related interventions, policy or programmes to maximise the sensitivity of the search (see S1 Table for search strategy). Comprehensive searches of Pubmed, Embase, and *The Cochrane Library* (CLIB) were undertaken to identify systematic reviews and meta-analyses evaluating any alcohol prevention intervention implemented at a community or population-level reported between 2006 and 2017. An initial search was conducted in April 2016 and updated in July 2017 to ensure it was current. We scanned reference lists of included articles and contacted experts in the field to ensure all relevant systematic reviews were identified. The search was not limited by publication status or language and eligible articles were translated by a professional service when required.

### Inclusion criteria

#### Study design

We included systematic reviews (with or without meta-analyses) which we defined as syntheses that: 1) collate evidence that fits pre-specified eligibility criteria in order to address a specific research question; 2) report explicit systematic methods; and 3) include a comprehensive search strategy of at least two electronic databases to identify primary studies. Reviews of either experimental or observational studies, or both, were eligible, but reviews of qualitative studies only were excluded.

#### Intervention

We defined alcohol control and public health interventions as those prevention interventions or policies which are implemented at a population or community level, and can be conceivably incorporated into legislation. Any of the following interventions included as one of the WHO recommendations for governments to address the harms due to alcohol consumption [5] were eligible:

- Drink-driving policies and countermeasures.
- Availability of alcohol.
- Marketing of alcoholic beverages (including online and social media platforms).
- Pricing policies.
- Reducing the public health impact of illicit alcohol and informally produced alcohol.
- Community action.
- Reducing the negative consequences of drinking and alcohol intoxication.

Where overlap existed in categorising an intervention (e.g. community-based programmes to reduce drink driving) we preferentially selected one category under which to report the review and report this in the text.

Prevention programmes focused within educational settings (universities, colleges or schools) and prevention and treatment programmes provided within the healthcare sector were excluded as delivery of interventions in these settings is less likely to be the focus of legislation. Interventions provided at an individual level only were also excluded.

#### Review selection and assessment of review quality

Two investigators (NS and CP) independently selected potentially eligible systematic reviews from the records yielded in the search. The full-text article was obtained for selected reviews and for those where these was uncertainty. Both investigators independently applied a standardised form to each article to assess eligibility according to the inclusion criteria: 1) systematic review design; 2) included prospective studies; 3) general population (whether the intervention was amenable to legislation); and 4) alcohol consumption or related harms measured (see S1 File for eligibility form). Any disagreements were resolved through discussion. Both investigators independently evaluated the methodological quality of each eligible systematic review using the ROBIS tool which permits classification of the conduct of reviews as at *high*, *low* or *uncertain* risk of bias. Risk of bias is dependent on an evaluation of review validity in four domains: 1) study eligibility criteria; 2) identification and selection of studies; 3) data collection and study appraisal; and 4) synthesis and findings [4]. Any disagreements in the ratings were resolved by discussion, noting ambiguities in the ROBIS tool where this was present. As ROBIS is a new tool we contacted the development team on several occasions for clarifications and advice in addition to the available guidance. We calculated the inter-rater agreement for the ROBIS tool for each of the four domains and for the overall risk of bias assessment using Cohen’s kappa coefficient (for three categories: *high*, *low* and *unclear* risk of bias). We interpreted the agreement using the Altman scale [7]. To address a conflict of interest for a single review where both investigators of this umbrella review were also authors [8], ROBIS was conducted by a senior researcher with expertise in ROBIS but not involved in this umbrella review.

### Data synthesis

NS extracted data into an MSExcel spreadsheet and both NS and CP grouped reviews by WHO intervention category [5]. The review characteristics and methodological quality were tabulated within these categories. Where data was missing or unclear we contacted review authors to obtain further information. In order to comprehensively summarise the evidence base for each intervention type, we planned to select the most recent review which conformed to a low risk of bias as measured by the ROBIS tool and to categorise the arising evidence based on the estimates of effect and the overall quality assessment. However, as so few reviews were rated at low risk of bias (N = 10), we instead assessed the overlap between review foci and where overlap existed, we selectively report on the review of high quality, or if no high quality reviews exists we report on the consistency of findings between the most recent reviews (published since 2015 inclusive). Where no overlap of review foci existed, we report on each review which evaluated a discrete intervention within the over-arching WHO categories. We judged the effectiveness for each intervention as: 1) beneficial, 2) possibly beneficial, 3) no benefit, 4) harmful, or 5) uncertain. This judgement was based on the direction of the effect of the intervention and a further three dichotomous variables and two qualitative variables. Reviews were awarded a point if 1) a quality assessment of the included studies was conducted, 2) if effects were consistent between primary studies and 3) if primary study quality was integrated into the overall results reported in the review through use of GRADE or a similar approach. For the qualitative variable of design of included studies, we weighted reviews which included randomised controlled trials and controlled prospective studies more than those including only uncontrolled observational studies. For effect size, we planned to weight a precise estimate with a narrow confidence interval derived from a meta-analysis more than an imprecise estimate, regardless of statistical significance. However, very few reviews included meta-analysis limiting the utility of this variable. Lastly, where applicable, we also considered the consistency of review findings where more than one review contributed data to the judgement. A review which met all or most of the conditions above and indicated a beneficial effect, was judged to be beneficial or probably beneficial and vice versa. Based on these judgements of effectiveness, we then assessed whether methodological revisions of the review were required and described the implications for primary research and/or policy implementation.

## Results

### Results of the Search

**Fig 1:**
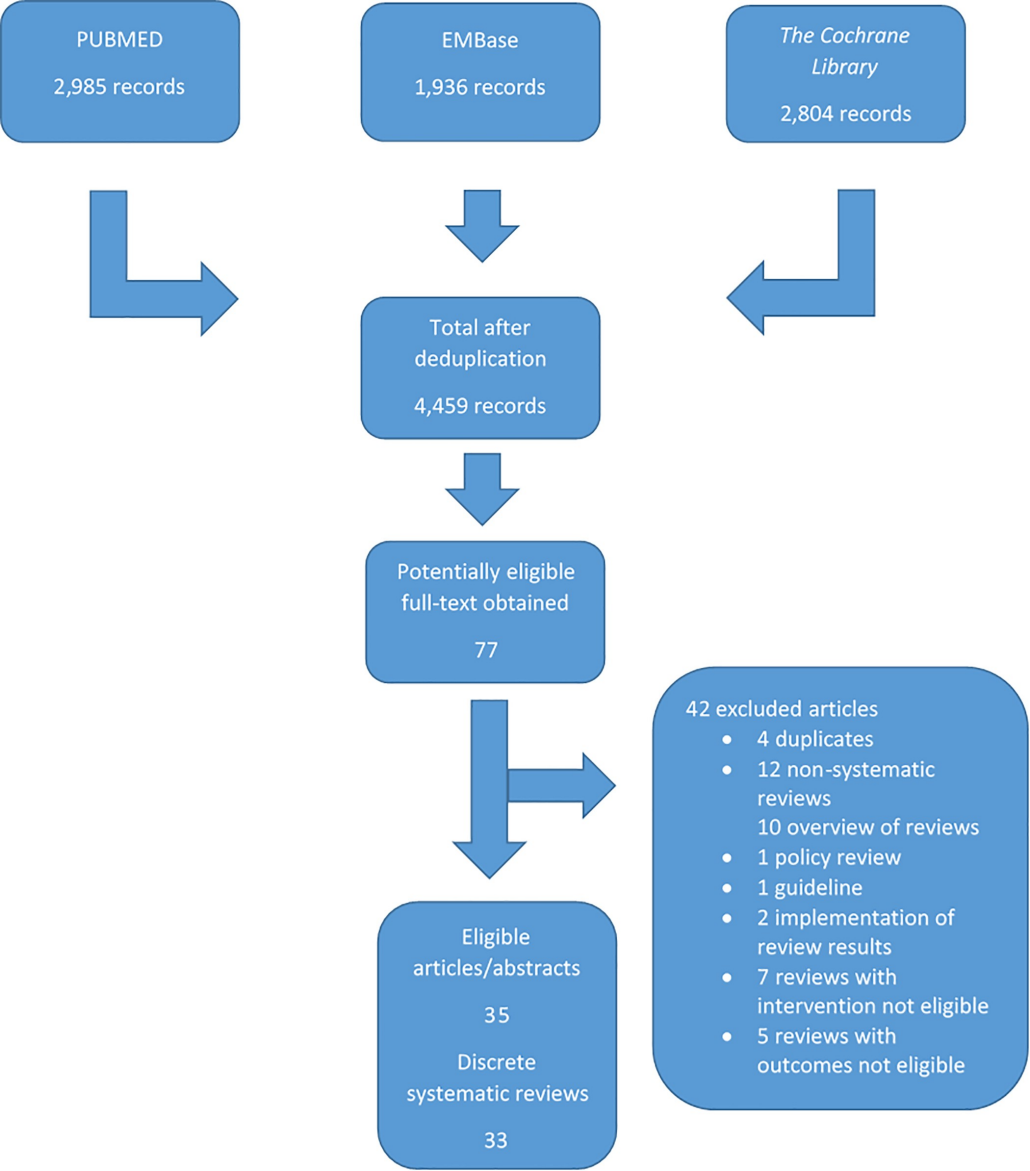
PRISMA Flow Diagram of 2016.

**Fig 2:**
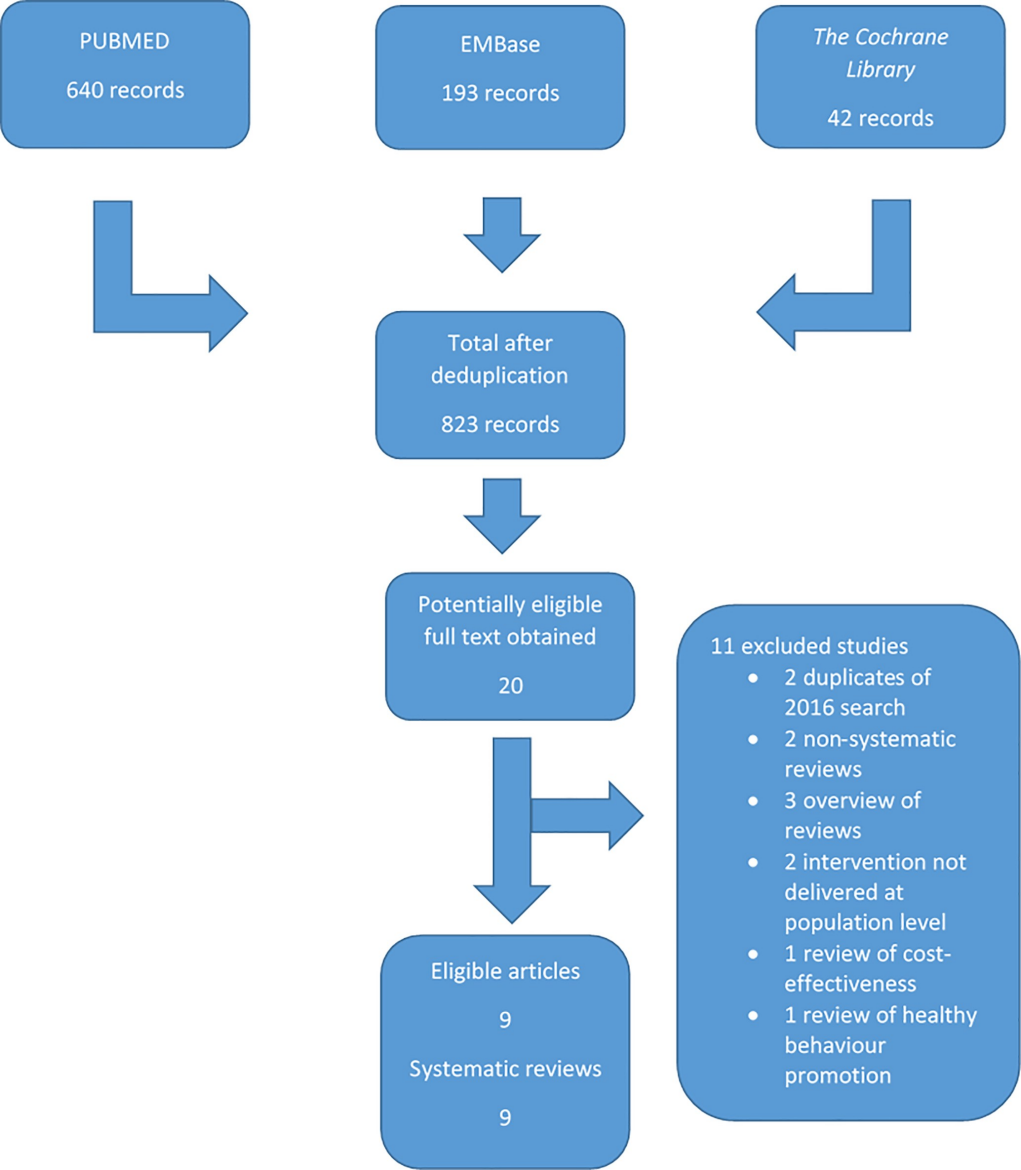
PRISMA Flow Diagram of 2017.

The April 2016 search retrieved 4,459 records from Pubmed, Embase and *The Cochrane Library* following electronic deduplication. From these, we identified 77 records as potentially eligible and obtained the full-text articles of which 35 articles reported on 33 discrete reviews. We identified a further nine discrete reviews following the July 2017 search, resulting in a total of 42 eligible systematic reviews for inclusion in this overview (See Figs 1 and 2 for search results). Seven articles required translation in order to assess eligibility: one from German [9,10], four from Portuguese ([11–14], one from French [15], and one from Spanish [16]. A list of excluded studies is available on request.

### Characteristics of the included systematic reviews

#### Intervention categories

Thirty-four reviews evaluated a single alcohol control intervention category with the remaining eight reviews evaluating multiple intervention categories. Restricting availability of alcohol was evaluated in 15 reviews, drink-driving policies were evaluated in 10 reviews, pricing policies were evaluated in 10 reviews, interventions to reduce the negative consequences of drinking and alcohol intoxication were evaluated in seven reviews, marketing policies were evaluated in three reviews, community action was evaluated in three reviews, and a harm reduction approach and reducing the use of illicit alcohol were each evaluated in a single review respectively. Sub-categories of interventions within the broad WHO categories can be seen in Table 1.

**Table 1:** Included studies and ROBIS quality assessment.

ID	Publication year	Number and type of studies	Publication limits	Language limits	ROBIS Domains				
					1	2	3	4	Overall
**INTERVENTIONS TO REDUCE DRUNK DRIVING**									
*Blood alcohol concentration limits*									
Araujo	2016	2 LS	No	No	Low	Unclear	Low	High	High
Aguilera	2014	1 ITS, 1 TS, 1 BA	Yes	Yes	High	High	High	High	High
Li	2014	1 CBA, 1 UBA	Yes	No	Low	Unclear	High	High	High
*Police Patrols*									
Erke	2009	40 observational studies	Unclear	Unclear	High	High	Unclear	Low	High
Goss-Cynthia	2008	1 RCT, 8 CBA, 14 CITS, 6 ITS, 3 CBA/ITS	No	No	Low	Low	Low	Low	Low
*Mass Media Campaigns*									
Yadav	2015	9 CITS, 7 ITS, 3 CBA	Yes	Yes	High	Low	Unclear	High	Low
Bergen	2014	4 CITS, 1 UBA	Yes	Yes	Low	Unclear	High	High	High
*Occupational Screening of Drivers*									
Aguilera	2014	1 UBA	Yes	Yes	High	High	High	High	High
Cashman	2009	2 ITS	No	No	Low	Low	Low	Low	Low
*Licence revocation*									
Araujo	2016	2 LS	No	No	Low	Unclear	Low	High	High
*Ignition locks*									
Elder	2011	1 RCT, 14 observational studies	No	Unclear	High	High	Low	Unclear	High
*Multicomponent programmes*									
Shults	2009	Reported under Community Action Interventions							
**COMMUNITY ACTION INTERVENTIONS**									
Muhunthan	2017	18 observational studies ((UBA, ITS and CS but not enumerated)	Yes	No	Low	Low	High	High	High
Jones	2011	7 RCTs, 7 CCTs, 6 UBA, 3 cohort analytical studies, 5 ITS	Yes	No	Low	Unclear	High	Low	High
Shults	2009	2 RCT, 2 CITS, 2 CBA	Yes	Yes	High	Low	Low	Low	High
**INTERVENTIONS TO REDUCE UNRECORDED ALCOHOL**									
Lachenmeier	2011	< 30 observational studies but no further details provided	Unclear	No	Low	Unclear	High	High	High
**MARKETING**									
Stautz	2016	7 RCTs	No	No	Low	Low	Low	Low	Low
Siegfried	2014	1 RCT, 3 CITS	No	No	Low	Low	Low	Low	Low
**REDUCING THE NEGATIVE CONSEQUENCES OF ALCOHOL**									
*Sporting settings*									
Kingsland	2016	3 RCT	No	No	Low	Low	Low	Low	Low
Priest	2008	0 studies	No	No	Low	Low	Low	Low	Low
*Targeting pregnant women*									
Crawford-Williams	2015	2 RCT, 2 repeated measures CS, 3 retrospective CS	Yes	Yes	High	High	Unclear	Low	High
*Labelling (includes low-alcohol and warning labels)*									
Shemilt	2017	1 CCT	No	No	Low	Low	High	Low	Low
Scholes-Balog	2012	10 CS	Yes	NR	Low	Unclear	High	Unclear	High
*Portion size (reduced)*									
Hollands	2015	0 studies	No	No	Low	Low	Low	Low	Low
*Interventions in and around licenced premises*									
Brennan	2011	5 RCT, 10 controlled observational studies	No	No	Low	Unclear	Unclear	High	High
**LIMITING AVAILABILITY (HOURS AND DAYS OF SALE, OUTLET DENSITY)**									
Nelson	2017	29 survey & registry data	Yes	Yes	High	High	High	High	High
Sanchez-Ramirez	2017	26 not clearly reported	Yes	Yes	High	Low	High	High	High
Wilkinson	2016	4 CITS, 6 ITS, 4 CBA, 3 BA, 3 DD, 1 'quasi-experimental'	Unclear	Yes	Unclear	High	High	High	High
Aguilera	2014	1 ITS	Yes	Yes	High	High	High	High	High
De Jong	2014	Not reported	Unclear	No	High	High	High	High	High
Wilson	2014	1 CITS, 1 ITS, 9 CS, 4 LS	Yes	No	Unclear	Unclear	High	Unclear	High
Bryden	2012	4 LS, 5 BA, 17 CS	No	No	Low	Low	Unclear	Low	Low
Hahn	2012	16 ITS, 1 LS	Yes	Yes	High	Low	Low	Unclear	High
Jones	2011	7 CT, 5 ITS, 3 LS, 6 BA,	Unclear	Yes	Low	Unclear	High	Low	High
Korszak	2011	1 LS, 2 CEA	No	Yes	Low	Low	Unclear	Low	Low
Rammohan	2011	10 CITS, 1 ITS	Yes	Yes	High	Low	Unclear	Low	High
Hahn	2010	1 ITS, 1 CBA, 2 LS, 10 UBA, 1 CS,	Yes	Yes	High	Low	Low	Low	High
Middleton	2010	9 CITS, 1 CBA, 1 BA	Yes	Yes	High	Low	Low	Low	High
Popova	2009	59 not clearly delineated	No	Unclear	Unclear	Low	High	Unclear	High
Spoth	2008	10 observational studies	Unclear	No	Low	Unclear	Unclear	High	High
**TAXATION AND PRICING**									
*Consumer Tax*									
Nelson	2017	29 survey & registry data	Yes	Yes	High	High	High	High	High
Nelson	2016	45 survey & registry data & hospital records	Yes	Yes	High	High	High	Low	High
Nelson	2015	56 econometric studies, 5 natural experiments, 6 field studies	Yes	No	High	Unclear	High	Low	High
Li	2014	3 ITS, 1 CBA, 4 BA	No	Yes	Low	Unclear	High	High	High
Korszak	2011	1 LS, 2 CEA	No	Yes	Low	Low	Unclear	Low	Low
Elder	2010	73 observational studies (ITS and panel) not clearly reported	Yes	Yes	High	Unclear	Low	Low	High
Wagenaar 09 & 10, Tobler	2010	115 observational, not clearly delineated	Yes	Unclear	High	High	High	High	High
Wilson	2014	1 CITS, 1 ITS, 4 LS, 9 CS	Yes	No	Unclear	Unclear	High	Unclear	High
*Retailer Tax*									
Wright	2017	1 case study	Yes	No	High	Low	High	High	High
*Minimum unit pricing*									
Boniface	2017	8 ITS, 1 CT, 9 CS, 13 modelling	Yes	No	High	Unclear	Unclear	Unclear	High

Abbreviations: RCT–randomized controlled trial; CBA–controlled before-after study; BA–(uncontrolled) before-after study; LS–longitudinal study; CS–cross-sectional study; CITS–controlled interrupted time series; ITS–(uncontrolled) interrupted time series; CEA–cost-effective analysis; DD–difference in difference

#### Publication

The mean number of systematic reviews published per year was 4.2 (standard deviation (SD) = 1.6) with the greatest number of reviews (N = 6) published in each year of 2011, 2014 and 2017. No eligible reviews were published in 2007. The trend test for publication over time was R^2^ = 0.4936. Almost all eligible reviews were published in English, except for one in German [9] and one in Portuguese [11].

#### Types of studies included in the reviews

None of the systematic reviews limited inclusion criteria to randomised controlled trials. All sought to search for, and include, a broad range of study designs. However, most reviews identified only observational studies relevant to the focal intervention(s) (74%; 31/42). Of the nine reviews which also identified randomised controlled trials (RCTs), seven reviews identified three or less relevant RCTs. A review of interventions delivered within drinking environments identified seven relevant RCTs [17] as did a review of exposure to alcohol advertisements [18]. There was no reporting of the study design of included studies in two reviews.

#### Location of studies included in the review

Thirty-five reviews adopted a global focus when searching for primary studies, with five reviews limiting inclusion to studies from high-income countries and two limiting inclusion to specific countries viz. USA and a combination of Denmark, Finland, Hong Kong, Sweden and Switzerland. Three and two primary studies were identified from the upper-middle income countries of China and Brazil respectively, and a single primary study was identified from each of the upper-middle-income countries of Colombia, South Africa, Thailand and Mexico. No studies were identified from low or lower-middle income countries.

### Methodology of included systematic reviews

#### Electronic searches and grey literature

Together the 42 reviews searched for studies across 51 different electronic databases. The most common databases were Medline, PsychInfo and EMBase searched in 41, 27 and 24 reviews respectively. *The Cochrane Library* was searched in 16 reviews and the Web of Science in 13 reviews. The economic database, EconLit, was searched in eight reviews focused on pricing and taxation. The nursing database, CINAHL, and the social science database, Sociological abstracts and social services abstracts, were searched in eight and seven reviews respectively.

Nineteen reviews stated that the search was not limited by publication status and included additional means to identify studies from the grey literature. Four reviews did not report whether or not publication status was a limitation, of which two provided further details suggesting that additional unpublished studies were included. Methods to identify unpublished studies included conducting ancestry reviews of reference lists (N = 11), and searching relevant websites (N = 7), Google Scholar (N = 6), and trials registries (N = 3). Four reviews described conducting hand-searching which included 1) searching journals not indexed in the major databases and available only in specialist addiction libraries [19], 2) searching an extensive collection belonging to the review authors [20], 3) hand-searching the journal *Traffic Injury Prevention*, publications of the International Council on Alcohol, Drugs, and Traffic Safety’s Working Group on Alcohol Ignition Interlocks, and the proceedings of the International Symposia on Ignition Interlocks [21], and 4) hand-searching all issues of the journals *Traffic Injury Prevention* and *Accident Analysis and Prevention* [22].

#### Language limitations

Eighteen reviews reported limiting inclusion to studies reported in English. Sixteen reviews reported that inclusion was not limited to studies reported in English: of these, three reviews stipulated that inclusion was limited to studies reported in English or German [9], English or Chinese [23], and English, Portuguese or Spanish [11]. It was not possible to determine language limitations in the remaining eight reviews.

### Risk of Bias in included systematic reviews

#### ROBIS findings

The four and overall ROBIS domains are presented in Table 1. Ten reviews (24%) were assessed as at low risk of bias, of which five were Cochrane systematic reviews and one reported applying Cochrane review methods but was not published as a Cochrane review.

We classified 75% (31/41) of reviews as at high risk of bias (we were unable to categorise one review due to the poor quality of reporting). Of these, eight conformed to the rigorous methods outlined by the United States non-federal *Task Force on Community Preventive Services (*Task Force) [24], but were classified as high risk due to limiting the search to English language publications and, in one such review [25], despite reporting limitations in the study quality, the interpretation of the overall evidence was categorised as ‘strong’. In the remaining 23 reviews which did not conform to Cochrane or Task Force methods, the reasons driving our decision to classify the risk of bias as high included alone or in combination: 1) publication and language limitations; 2) study selection and/or data extraction conducted by a single investigator; 3) lack of quality assessment of included studies and lack of integration of quality assessment into the results; and 4) contradictions between the presented data and the authors’ conclusions.

#### Agreement between investigators

Inter-rater agreement for the ROBIS overall risk of bias was calculated for the 40 reviews which were rated by both investigators. The Cohen’s kappa coefficient for the overall risk of bias was 0.34 interpreted as fair agreement using the Altman benchmark scale [7]. For Domains 1 to 4 the coefficients were 0.51 (moderate), 0.38 (fair), 0.65 (good), and 0.51 (moderate) respectively.

### Evidence for alcohol control policy interventions

#### Community action interventions

Three reviews evaluated qualitatively different multicomponent interventions which were all delivered in the community [17,25,26]. None of the reviews conducted meta-analyses and all were rated as high risk of bias.

Community mobilisation: In 2009, Shults et al. [25] evaluated the effects of community mobilization on reducing alcohol-impaired driving specifically. Community mobilization was defined as organization and activation of a community to address local problems as part of multicomponent programmes which included a combination of responsible beverage service activities, enforcement of minimum legal drinking age laws, controlling alcohol outlet density, sobriety checkpoints, public education and media advocacy. The review authors concluded that evidence was ‘strong’ in favour of multicomponent programmes when implemented with community mobilization for reducing alcohol-related crashes, despite identifying several shortcomings within the studies and stating that there was a lack of unequivocal evidence. Given this contradiction, combined with a high risk rating on ROBIS, our assessment was that such programmes are *possibly beneficial* and that a full update and revision of the review is required. In the interim implementation of such programmes should include monitoring and evaluation.

Interventions implemented in the drinking environments: A 2011 review by Jones et al. [17] evaluated interventions implemented in drinking environments and was rated as at high risk of bias primarily due to a lack of clear reporting on duplicate and independent data extraction and publication limitations. The authors integrated the quality assessment of included studies with the numerical results and concluded that multicomponent intervention programmes combining community mobilisation, responsible beverage server training, house policies and stricter enforcement of licencing laws are possibly effective in reducing assaults, traffic crashes and underage sales, but there is less certainty for other interventions including patron-targeted interventions and stand-alone server training. Our assessment of their reported results confirmed their findings as *possibly beneficial* in favour of multicomponent interventions. The review requires updating with more recent studies and until such time, implementation of such programmes should be done within a monitoring context.

Indigenous community-led legal interventions to control alcohol: The 2017 review by Muhunthan and colleagues [26] evaluated 18 observational studies of indigenous community-led legal interventions to control alcohol and concluded that these can be effective in improving health and social outcomes. Given the high risk of bias in this review due to a lack of quality assessment of included studies and publication limitations, our assessment was that evidence is currently *uncertain* and that a full review revision with both quality assessment and integration of results using a GRADE approach is required prior to implementation and to inform future research studies.

#### Interventions to reduce unrecorded alcohol

A single 2011 review without a meta-analysis evaluated the effects of policies to reduce the impact of unrecorded alcohol use and concluded that there is currently no clear evidence base on the effectiveness (or cost-effectiveness) of available policy options [20]. The review included less than 30 studies, described as ‘mostly observational’, and no quality assessment of included studies was undertaken. The ROBIS rating was high risk of bias. Our assessment concurred with the authors that evidence remains *uncertain*, and the review requires updating and revision with a quality assessment and integrated approach, to determine the need for, and design of, future controlled studies and implementation strategies within monitoring contexts.

#### Regulation of marketing beverages

Two reviews with meta-analyses met the inclusion criteria to evaluate reducing or banning the marketing of alcohol and its effects on alcohol consumption through an interventional lens [8,18]. Both were rated as at low risk of bias, but assessed consumption at different time periods. The 2014 review by Siegfried *et al*. included three controlled interrupted time series studies of state- and provincial-wide banning of alcohol advertising in combinations of print, electronic and billboard media, and long-term consumption and concluded that due to methodological deficiencies in the dated studies (1976, 1980 and 1991), the current evidence base was uncertain. As co-authors of this review, we acknowledge that the review requires an updated search, and in the interim implementation of country-wide banning of alcohol advertising should be implemented with ongoing monitoring to allow for adequate analyses.

The 2016 review by Stautz *et al*. considered immediate alcohol consumption following exposure to television or movie alcohol advertising in a meta-analysis of seven laboratory-based RCTs. The review authors conclude that viewing alcohol advertisements may increase immediate alcohol consumption by a standard mean deviation of 0.20 (95% CI: 0.05, 0.34), interpreted as an increase of 1.57 (95% CI: 0.39, 2.67) alcohol units [18]. Given the low risk of bias and the integration of the results with a thorough quality assessment, we agree that restricting alcohol advertising is *possibly beneficial* in the short-term (limited to a few hours). Additional controlled studies are required in non-student populations to evaluate generalizability prior to scale-up.

#### Availability

Fifteen reviews evaluated availability interventions including licencing restrictions on trading times, minimum drinking age, outlet density and distance, retail privatization and dram shop liability with significant overlap between reviews [9,11,17,27–38]. Two reviews were rated as low risk of bias (both published before 2015) and of these, we report the more recent review preferentially.

Bryden et al. published a well-conducted review in 2012 and state that overall the narrative results for restricting availability were inconclusive but that higher outlet density may lead to increased alcohol use. Our assessment concurs that the evidence is *uncertain* for licencing restrictions (including banning sales, and making changes to the hours, days and volumes of alcohol sales) but that reducing outlet density is *possibly beneficial*. The review requires updating with more recent studies and additional longitudinal primary research is required. Implementation of policies should undergo monitoring and evaluation.

#### Drink-driving interventions

Nine reviews evaluated six interventions aimed at addressing drunk driving [11,21–23,25,39–43].

Blood alcohol concentration limits: Three reviews evaluated the effects of blood alcohol concentration (BAC) limits on traffic injuries [11], mortality from motorcycle crashes [42], and levels of BAC in Chinese drivers respectively [23]. All were assessed as at high risk of bias. None of the reviews included overlapping primary studies or outcomes and we therefore report on all three review findings briefly.

The 2014 review focused on all traffic injuries and reported that no beneficial effects were found unless the BAC limit was combined with other interventions [11]. The 2016 review specific to the effects of BAC limits on motorcycle crash mortality included two longitudinal studies and reported the results to be inconsistent but concluded that it was a potentially effective intervention [42]. The China-specific review included a very large 2009 controlled-before-after study of enhanced BAC enforcement among 32,101 drivers which reportedly found that the intervention was successful in decreasing driver BAC (but traffic injury was not measured as an outcome) [23].

Given the above inconsistencies in review results, the differences between the included studies and the high risk of bias observed in all three reviews, we assessed the overall effect of BAC limits (when not combined with other interventions) as *uncertain*. A single over-arching review is required with an updated search for primary studies, an overall quality assessment, and integration of this with the results. In the interim implementation should be conducted in a monitoring environment and additional controlled studies are required.

Police patrols: Two reviews, published in 2008 and 2009, evaluated the effectiveness of police patrols in reducing alcohol-related crashes [39,40]. Given that both reviews are out of date, we selected the 2008 Cochrane review as the primary data source given its low risk of bias rating. The authors included 1 RCT and 31 observational studies and concluded that there is consistent evidence that police patrol programmes reduce traffic crashes and fatalities, but noted that due to limitations in study quality and data analysis, the evidence was supportive but not unequivocal [40]. Our assessment was that police patrols are *possibly beneficial*. The review requires updating and we agree with the authors that further well-designed controlled studies are required. Implementation should occur in a monitoring environment.

Mass media campaigns: Two reviews focused on mass media campaigns, a 2014 review evaluated the effects on increasing awareness of publicized sobriety check-points [43] and a 2015 review assessed the effects on alcohol-impaired driving and alcohol-related crashes [41]. The 2015 review, an update of a previous review [44], was rated as at low risk of bias so we selected to summarise these results. The review conducted a meta-analysis which did not show any improved risk of alcohol-related injuries or fatalities from the intervention (RR = 1.00, 95% CI = 0.94–1.06). The review authors note the heterogeneity among the media campaigns, in the methods used and in the outcome measurements, as well as the observed statistical heterogeneity in the pooled results. Given these concerns, the authors state that they cannot conclude that media campaigns reduce the risk of related alcohol-related crashes or injury. Our assessment is that the evidence is *uncertain* and we concur with their recommendation that better controlled studies should be conducted, especially with respect to newer media methods.

Screening of occupational drivers: Two reviews evaluated testing occupational drivers for alcohol to prevent injury or work-related effects. A 2009 Cochrane review, rated as at low risk of bias, concluded that there was insufficient evidence for or against alcohol testing [22]. A 2014 review, rated as high risk of bias, searched for studies published after 2006 and identified a single uncontrolled before-after study and reported that fatalities were reduced by 80% in truck drivers and by 41% in other drivers involved after compulsory alcohol testing of drivers [11]. Given the inconsistency in these results and the need to update and combine the data synthesis, we assessed the current evidence as *uncertain* and advocate for an updated and revised review to establish the evidence base and to determine if there is a need for conduct of further controlled studies or ongoing monitoring.

Administrative licence revocation: A 2016 review, rated as at high risk of bias, included two longitudinal studies (see also under BAC limits) that considered administrative licence revocation (e.g. if individuals refuse a blood alcohol test) to reduce motorcycle crashes specifically [42]. The studies found inconsistent results and the authors conclude that the intervention is potentially effective, despite reporting that there is doubt whether the intervention has a significant effect on crash and injury rates. Given the risk of bias, the limited number of studies and the inconsistency reported in results, we assess the evidence as *uncertain* and recommend the review be revised with integration of the study quality into the conclusions. Implementation should be done with ongoing monitoring.

Ignition Locks: A 2011 review updated a 2004 Cochrane review [45] with grey literature searching (no revised electronic database searching was conducted) and evaluated the effects of ignition interlocks for preventing alcohol-impaired driving and crashes in drivers convicted of driving under the influence of alcohol [21]. The review was rated as at high risk of bias and the authors concluded that there is ‘strong’ evidence that installation is associated with large reductions in re-arrest rates for alcohol-impaired driving but evidence was insufficient regarding the effect on alcohol-related crashes. We noted the large effect reported consistently across primary studies but given that limitations of the observational studies were not adequately integrated into the results and the high risk of bias rating, we assessed the evidence as *possibly beneficial* and a revised, and updated review is required.

#### Reducing the negative consequences of drinking and alcohol intoxication

Seven reviews appraised five interventions aimed at reducing the negative consequences of drinking and alcohol intoxication. None included meta-analyses.

Interventions for disorder and severe intoxication in and around licenced premises: A 2011 review was rated at high risk of bias (primarily due to a lack of clear reporting regarding review methodology) [46]. Interventions included responsible beverage server training, server violence prevention training, enhanced enforcement of licencing regulations, multi-level interventions, licensee accords, and a risk-focused consultation. The review authors found that server training courses designed to reduce disorder show potential, but lack evidence to support reducing intoxication. We concur with the authors’ conclusions that the evidence base is *uncertain* overall and *possibly beneficial* for server training courses. However, the review requires updating with recent studies and revision incorporating a GRADE-like approach.

Alcoholic beverage labels: Two reviews evaluated labels on alcoholic beverages: a 2012 review focused on the effects of alcohol warning labels in adolescents [47] and a 2017 review evaluated the effects of labels stating ‘low alcohol’ [48].

The review of warning labels was rated at high risk of bias. No quality appraisal was conducted, although the authors did consider the limitations of the cross-sectional nature of the studies on the overall evidence. They concluded that warning labels may show increased awareness among adolescents, but have little effect on individual beliefs of risk or on alcohol-related behaviours. Our assessment is that the evidence is *uncertain* and the review requires updating and a revision with a validated quality assessment of studies and integrated GRADE approach. In the interim, the effects of implementation should be monitored.

The 2017 review of ‘low alcohol’ labelling employed Cochrane methods and included a single non-randomised cross-over controlled trial [48]. The review concluded that evidence is uncertain. We noted that the evidence is current and of a high quality and agree with the authors’ conclusions that the evidence is *uncertain* and that there is an urgent need to conduct well-designed controlled studies of this intervention.

Portion size: A 2015 Cochrane review evaluated the effect of portion size on alcohol consumption [49]. This review was rated as at low risk of bias. No eligible studies were identified and as such, the authors identify a lack of evidence. We assessed the evidence as current and concur with the authors’ conclusion that well-designed controlled studies are required prior to policy implementation on this issue.

Interventions in sport settings: Two reviews evaluated policy interventions delivered in sports settings, both were rated as at low risk of bias and no meta-analyses were conducted [50,51]. The 2016 review was conducted according to Cochrane methodology and identified two cluster RCTs of policy interventions [51]. The review authors conclude that the effects are inconsistent and that given the paucity of studies in this area, more well-conducted controlled studies are required. We assessed the evidence as current and concur with the authors’ conclusion of the evidence as *uncertain*.

Public health interventions specific to pregnant women: A 2015 review of public health interventions aimed at reducing alcohol consumption and/or increasing knowledge among pregnant women was rated as at high risk of bias. The authors considered the impact of the quality of studies on the results, concluding that there is little evidence available on the effectiveness of pregnant-specific interventions [52]. We assessed the current evidence as *uncertain*, and concur with the authors’ conclusions that well-conducted controlled studies are required.

#### Taxation and pricing

Ten reviews reported in 11 articles and one abstract evaluated the effects of taxation and pricing, or both, on alcohol consumption or related harms [9,23,36,38,53–59]. Several reviews had specific foci including youth [9], China [23], intimate partner violence [36] and binge drinking [55]. All, except the review on youth, were rated at high risk of bias.

Increased pricing and taxation: We selected to report in full on the four reviews conducted since 2015 given that all were at high risk of bias. Three reviews, all authored by Nelson *et al*., were conducted in 2015 [55], 2016 [54] and 2017 [38] and utilised data from the same database supplemented with discrete searches to evaluate the effects of price and taxation. The reviews were limited by including only English publications, a lack of duplication of data extraction, and no assessment of study quality or integration thereof into the results.

The 2015 review included 56 econometric studies and evaluated the consistency and significance of findings across studies, concluding that binge drinkers are not highly responsive to taxation or pricing. The responsiveness was not quantified and we conclude that for binge drinking specifically the evidence remains *uncertain*.

For the 2016 and 2017 reviews conducted to assess harms in nine countries and consumption in five countries, 45 and 29 studies were included respectively. The authors found considerable diversity and inconsistency in the research base for price and taxation to reduce alcohol consumption and harms. They state that they do not argue that prices have no effect on alcohol consumption, but that the effects depend on temporality, location, and population factors. We assessed the evidence as *possibly beneficial*, and recommend revision with adequate quality assessment, meta-analysis and regression where possible to account for the potential confounders, and integration of the overall quality into the interpretation of the results.

Price only (minimum unit pricing): A 2017 review focused on minimum alcohol unit pricing (MUP) excluding studies on taxation and elasticity [58]. The review appraised the data using Bradford-Hill causality criteria and integrated the quality assessment into the final conclusions. Meta-analysis was not conducted. It was rated at high risk of bias due to uncertainty regarding duplication of screening and data collection and limitations to English language. The authors conclude that given the lack of controlled studies, it is highly probable, but not definite, that MUP reduces alcohol consumption and alcohol-related harms. We determined the evidence to be *possibly beneficial* and recommend that further implementation of MUP occur within a monitoring environment.

Tax on retailer: A 2017 review investigated taxation on retailers and manufacturers of unhealthy food products. The review identified 102 studies for inclusion [59], but only a single case study of taxation of large alcohol and tobacco retailers based on the value of their premises. The study reported that revenue raised was predictable and above government expectations, but the impact on alcohol-related consumption was not evaluated. The review authors conclude that retail taxation above 20% on unhealthy food products in general reduces consumption, but the evidence base for alcohol is a single study with no evaluative component. We assessed the evidence for alcohol as *uncertain*. The review requires revision with conduct and integration of quality assessment into the final results, and additional controlled studies are required with implementation within a research/monitoring context.

## Discussion

This overview of systematic reviews provides a comprehensive summary of the last decade of synthesis research into alcohol control policies and interventions. With few exceptions, the quality of systematic reviews of alcohol control research is characterised by inadequate methodology and both reporting and review conduct fail to meet acceptable PRISMA standards. The overview indicates that evidence remains uncertain for many interventions but is possibly beneficial for the following control measures: 1) community mobilization; 2) multicomponent interventions delivered in the drinking environment; 3) restricting alcohol advertising; 4) restricting on- and off-premise outlet density; 5) police patrols and ignition locks to reduce drink driving; and 6) increased price and taxation including minimum unit pricing.

Our assessment of the quality of the systematic reviews using ROBIS is dependent on the reporting of the reviews. Conduct of a review may not be fully reflected in an article due to imposed journal word count limitations resulting in an inaccurate ROBIS assessment of poor quality. However, it is likely that our ROBIS assessments were largely a true reflection of the limited quality given that few reviews reported duplicate screening and data extraction, most were limited to English and published studies, and very few integrated the risk of bias in the primary studies into the overall findings. Poor reporting may arise from authors’ and journal editors’ lack of knowledge about optimal reporting standards for systematic reviews as outlined in PRISMA [60]. We were not able to verify review methods as none of the reviews had been registered on PROSPERO, a publicly-accessible platform for prospective registration of review protocols [61]. Future reporting of reviews of alcohol control should conform to the PRISMA standards to ensure accurate quality assessment and authors should consider prospective registration of the review protocol to ensure transparency.

Several tools exist to assess the quality of the conduct of a systematic review including AMSTAR-2 [62] and the ROBIS tool [4]. The ROBIS tool was developed in 2016 and provides a judgement-based domain approach combined with an evaluation of the relevance of the included studies to the review question, and the degree to which the reviewers avoid emphasizing statistical significance. Our inter-rater agreement was fair to moderate with agreement lowest when rating the second domain focused on identification and selection of studies, and the domain rating the overall risk of bias. Buhn *et al*. evaluated ROBIS across four raters in 16 reviews [63]. In contrast to our findings, Buhn *et al*. found the highest level of agreement in the second domain and the lowest in the fourth domain of synthesis and findings. Buhn *et al*. identified that previous experience with rating reviews was associated with higher levels of agreement between pairs of reviewers. The differences in interrater agreements within our study and compared to Buhn *et al*., may reflect differences in our experience of evaluating reviews, but may also reflect ambiguities in the ROBIS tool. Additional reliability studies are required to better articulate these so that ROBIS guidance can be optimised. Nonetheless, the process of rating reviews independently and then resolving differences through discussion provided a useful framework as a starting point for judging quality.

The field of alcohol control, and public health in general, is very context-specific. ROBIS recommends including experts in the field to ensure quality domains are rated appropriately, e.g. to determine if a topic search requires a global or a localised focus, or if language limits are appropriate to a country-specific legislation and are therefore not a source of bias. These context-specific issues created a tension when viewing the results through a global lens as our umbrella review aimed to inform the revision of the WHO Strategy, and may have contributed to lower inter-rater agreement.

Despite the diversity and range of alcohol control research, the field is homogenously lacking in controlled studies. Few investigators utilise newer methods of policy evaluation including implementation science and pragmatic randomised trials. Alcohol control interventions tend to be complex, multi-faceted, and often multi-sectoral, which may be better suited to evaluation within an implementation research paradigm [64]. Implementation research seeks to explore effects in real-world conditions and outcomes can include acceptability, feasibility and costs [64]. Such an approach does not preclude controlled studies with controlled interrupted time series studies potentially providing a useful method to test policy changes when well-conducted with multiple time-points.

Outcome measurements were highly inconsistent across primary studies, limiting the opportunity for synthesis and meta-analysis within systematic reviews. This is not unique to the alcohol control field and is being addressed across healthcare research with the COMET (Core Outcome Measures in Effectiveness Trials) Initiative which aims to encourage researchers to agree upon and use the same, validated outcome measures across studies to facilitate data synthesis over time [65]. It would be expedient for those active in primary alcohol control research to consider development of a core outcome set specific to the field. Martineau et al. note that no single primary outcome adequately captures the full impact of population-level alcohol interventions, and suggest that lessons learnt from linking taxation interventions to a range of ascertainable outcomes may be extrapolated to other interventions [66].

In addition to poor reporting of applied methods, we noted poor reporting of the context-specific policy environment. As far as we are aware, no validated tool exist for generalizing from systematic reviews of alcohol control policy across space and time. While we identified interventions of possible effectiveness, we were not able to elaborate the contextual factors required in order to ensure effective implementation of these interventions. The TRAICE (Transparent Reporting of Alcohol Intervention ContExt) Checklist is available to investigators of primary studies to better report six policy context factors which may impact on effectiveness viz.: i) baseline alcohol consumption, norms and harm rates; ii) baseline affordability and availability; iii) social, microeconomic and demographic contexts; iv) macroeconomic context; v) market context; and vi) wider policy, political and media context [67]. None of the systematic reviews included in our overview reported that primary studies utilised TRAICE; however, as acknowledged by the authors, it requires further validation at primary study level and would require adaptation for use within a systematic review.

It is astounding to note that research arising from low- and lower middle-income countries is entirely absent from our overview, both at a review and a primary study level. This confirms our observation from more than a decade ago that evidence-based alcohol policy and associated research is lacking in low- and middle-income countries (LMIC) [68] and is consistent with the findings supporting the WHO Global Strategy [69]. The challenges to conducting evaluations of population-level interventions in resource-poor settings are many and include a lack of robust routinely-collected data and limited human capacity. However, a promising analysis of health policy and systems research (HPSR) in LMIC indicates that publications with a topic relevant to LMICs and an LMIC lead author continue to increase at a greater rate than the life and biomedical science topics in general [70]. The authors postulate that this is likely due to increased capacity for research within LMICs and support for publications surrounding large HPSR initiatives. There is clearly a need for investment in demonstration alcohol control projects in selected countries which have the technical expertise and financial resources to undertake high quality evaluations of population-level interventions to set the way for other LMICs to follow. The WHO has a role to play in supporting LMIC governments to implement evidence-based policies, to encourage research-based implementation, and to ensure monitoring data is appropriately collected and analysed.

This overview has significant strengths due to the comprehensive nature of the database search and inclusion of reviews regardless of language and translating these where necessary. Both reviewers independently screened studies, evaluated study eligibility, and conducted risk of bias assessments. Duplicate screening has been shown to maximize ascertainment of relevant studies [71]. However, inclusion of a third reviewer to resolve disagreements may further have strengthened this process. Comparison of interrater agreement with a third reviewer may also have clarified ambiguities in the ROBIS tool and provided additional pair-wise reliability data.

The overview is limited to the time period we searched and it is possible that systematic reviews of effective structural interventions prior to 2007 have been missed. However, given the comprehensive nature of the categories of intervention included in this overview, we believe this is unlikely. In the absence of a validated tool to integrate quality and review findings in an overview synthesis, we developed an *a priori* hierarchical decision-making algorithm to aid selection of individual reviews from which to extract effectiveness data. We further applied a systematised, transparent evaluation of dichotomous and qualitative review variables to inform our judgements of overall effectiveness of interventions. This compares favourably to a similar approach reported in the literature [72]. However, as outlined by McKenzie and Brennan, there is currently no guidance on how to integrate quality assessments into overviews when interpreting findings [73]. These methods require further application and testing in future overviews to inform reliability and validity.

It is reassuring that our findings of effectiveness are largely consistent with those outlined in the initial 2010 WHO Global Strategy [69] and the WHO SAFER alcohol control strategy launched in September 2018 [74]. A 2013 overview of systematic reviews, Martineau and colleagues [66] conducted quality assessment of the included reviews using the AMSTAR tool and categorised review findings by consistency of direction and significance of results. Review quality was not integrated into the assessments of the results, and the authors recommend that the results of the overview should only be considered in conjunction with the individual reviews and primary studies. This limits the utility of an overview to articulating the breadth of the evidence whereas policymakers require identification of the optimal choice of interventions. Our focus on integrating the quality of the review into interpretation of the review results may reflect more recent advances in the development of quality indicators aiming to provide policymakers with a more complete presentation of effectiveness. However, as stated previously, our approach to transparently integrate quality measures into the interpretation of review results requires future validation as it evolves. Future replication of the included systematic reviews using current comprehensive methods, such as led by the 3ie replication programme [75], would be informative to assess the impact of the potential biases identified in this overview.

## Conclusion

Our findings point to the need for more robust research methods in both systematic reviews and in primary research studies on the effectiveness of macro-level interventions to reduce consumption of alcohol and associated negative consequences. We categorised several interventions as of uncertain benefit in contrast to other literature that tends to propose alcohol policy measures with little attention to reporting doubts regarding effectiveness. Reporting of systematic reviews fails to meet internationally acceptable standards and review authors do not appear to utilise several available tools to improve both the conduct of their reviews and the reporting thereof. We believe this overview with its necessary focus on quality, further advances not only the alcohol control field, but also the methodology of integrating measures of quality into review syntheses.

Due to limitations in quality, we categorised many interventions as of uncertain benefit and for these, we advocate that additional primary controlled research is required prior to formulating policy recommendations. For the six interventions with evidence supporting their effectiveness, we recommend policy-makers ensure that their effects are monitored during implementation to build the evidence base in real-world settings. Research on alcohol control in low- and lower middle-income countries should be prioritised at a global level as policy requires rigorous evidence drawn not only from studies of the most robust design feasible, but also from those with greatest applicability to the local context and regulatory environment.

## Supporting information

S1 Table Search strategy for PUBMED. (DOCX)

S1 File Eligibility Form. (DOC)

S2 File PRISMA Checklist. (DOC)
