# Supplementary material for: Do alcohol control policies work? An umbrella review and quality assessment of systematic reviews of alcohol control interventions (2006 – 2017)
Source: PLoS One. 2019 Apr 10;14(4):e0214865. doi: 10.1371/journal.pone.0214865 (PMC6457561; doi:10.1371/journal.pone.0214865)
Supplement: S1 Table — (DOCX) [file pone.0214865.s001.docx]

**Table S1. Search strategy for PUBMED**

| Search | Query |
| --- | --- |
| [#10](https://www.ncbi.nlm.nih.gov/pubmed) | Search ((((#7 AND #8) NOT (animals[mh] NOT humans[mh])))) AND ("2016/04/01"[Date - Publication] : "2017/07/20"[Date - Publication]) |
| [#9](https://www.ncbi.nlm.nih.gov/pubmed) | Search ((#7 AND #8) NOT (animals[mh] NOT humans[mh])) |
| [#8](https://www.ncbi.nlm.nih.gov/pubmed) | Search (systematic[sb] OR systematic reviews[ti]) |
| [#7](https://www.ncbi.nlm.nih.gov/pubmed) | Search (#1 OR #2 OR #3 OR #4 OR #5 OR #6) |
| [#6](https://www.ncbi.nlm.nih.gov/pubmed) | Search (drink*[tiab] AND driv*[tiab] OR drunk driving[tiab] OR (driving[tiab] AND alcohol[tiab])) |
| [#5](https://www.ncbi.nlm.nih.gov/pubmed) | Search (drink*[tiab] AND (excess*[tiab] OR heavy[tiab] OR heavily[tiab] OR hazard*[tiab] OR binge[tiab] OR harmful[tiab] OR problem*[tiab])) |
| [#4](https://www.ncbi.nlm.nih.gov/pubmed) | Search (alcohol*[tiab] AND (drink*[tiab] OR beverage*[tiab] OR intoxicat*[tiab] OR abus*[tiab] OR misus*[tiab] OR risk*[tiab] OR consum*[tiab] OR excess*[tiab] OR problem*[tiab])) |
| [#3](https://www.ncbi.nlm.nih.gov/pubmed) | Search (alcoholic beverages[mh] OR alcoholic beverage*[tiab] OR wine*[tiab] OR beer*[tiab] OR spirits[tiab] OR liquor*[tiab]) |
| [#2](https://www.ncbi.nlm.nih.gov/pubmed) | Search alcohol related disorders[mh] |
| [#1](https://www.ncbi.nlm.nih.gov/pubmed) | Search alcohol drinking[mh] |
