# Supplementary material for: Do alcohol control policies work? An umbrella review and quality assessment of systematic reviews of alcohol control interventions (2006 – 2017)
Source: PLoS One. 2019 Apr 10;14(4):e0214865. doi: 10.1371/journal.pone.0214865 (PMC6457561; doi:10.1371/journal.pone.0214865)
Supplement: S1 File — (DOC) [file pone.0214865.s002.doc]

**ROBIS Umbrella review of alcohol control policies**

**Study eligibility form** __________________(study ID)

| Type of study | | | | | |
| --- | --- | --- | --- | --- | --- |
| Q1. Is the study design one of the following?    - Systematic Review  - Overview of reviews* | | Yes | | Unclear | No |
| * use references for cross-checking | | Go to  next question | | | Exclude |
| Included studies in the review | | | | | |
| Q2. Were the included studies?    - Randomised controlled trials | Yes | | | Unclear | No |
| - Controlled Before After studies  - Interrupted Time Series  - Longitudinal study | Go to  next question | | | | Exclude |
|  |  | | |  |  |
|  |  | | | |  |
| Intervention/comparison in the study |  | | |  |  |
| Q3. Was the intervention focused at a population level?  [Can the intervention be legislated?] | Yes | | | Unclear | No |
|  | Go to  next question | | | | Exclude |
| Outcome in the study |  | | |  |  |
| Q4. Did the review focus on alcohol consumption or alcohol-related harms as outcome(s)? | Yes | | | Unclear | No |
|  | Go to  next question | | | | Exclude |
| **Any other reasons for excluding study?**  **Specify:** | No | |  | | Yes |
|  | **Include**, subject to clarification of ‘unclear’ points | | | | Exclude |
| Final decision | **Include** | | **Unclear** | | **Exclude** |
